# An Increased Ratio of Glycated Albumin to HbA1c Is Associated with the Degree of Liver Fibrosis in Hepatitis B Virus-Positive Patients

**DOI:** 10.1155/2014/351396

**Published:** 2014-02-17

**Authors:** Hirayuki Enomoto, Nobuhiro Aizawa, Hideji Nakamura, Yoshiyuki Sakai, Yoshinori Iwata, Hironori Tanaka, Naoto Ikeda, Tomoko Aoki, Yukihisa Yuri, Kazunori Yoh, Kenji Hashimoto, Akio Ishii, Tomoyuki Takashima, Kazunari Iwata, Masaki Saito, Hiroyasu Imanishi, Hiroko Iijima, Shuhei Nishiguchi

**Affiliations:** ^1^Division of Hepatobiliary and Pancreatic Disease, Department of Internal Medicine, Hyogo College of Medicine, Mukogawa-cho 1-1, Nishinomiya, Hyogo 663-8501, Japan; ^2^Department of Gastroenterology and Hepatology, Nissay Hospital, Itachibori 6-3-8, Nishi-ku, Osaka 550-0012, Japan

## Abstract

*Background*. In hepatitis B virus- (HBV-) positive patients, the relationship between the metabolic variables and histological degree of liver fibrosis has been poorly investigated. *Methods*. A total of 176 HBV-positive patients were assessed in whom the ratios of glycated albumin-to-glycated hemoglobin (GA/HbA1c) were calculated in order to investigate the relationship with the degree of liver fibrosis. *Results*. The GA/HbA1c ratio increased in association with the severity of fibrosis (METAVIR scores: F0-1: 2.61 ± 0.24, F2: 2.65 ± 0.24, F3: 2.74 ± 0.38, and F4: 2.91 ± 0.63). The GA/HbA1c ratios were inversely correlated with four variables of liver function: the prothrombin time (PT) percentage (*P* < 0.0001), platelet count (*P* < 0.0001), albumin value (*P* < 0.0001), and cholinesterase value (*P* < 0.0001). The GA/HbA1c ratio was positively correlated with two well-known markers of liver fibrosis, FIB-4 (*P* < 0.0001) and the AST-to-platelet ratio index (APRI) (*P* < 0.0001). Furthermore, the GA/HbA1c showed better correlations with two variables of liver function (PT percentage and cholinesterase value) than did FIB-4 and with all four variables than did the APRI. *Conclusion*. The GA/HbA1c ratio is associated with the degree of liver fibrosis in HBV-positive patients.

## 1. Introduction

In patients with chronic liver disease (CLD), liver biopsy is the gold standard method to evaluate the degree of liver fibrosis [1]. However, a liver biopsy is a costly and invasive technique associated with a risk of complications. In addition, there can be sampling errors, because only 1/50,000 of the organ is used for the analysis [1]. Furthermore, it has been reported that there are inter- and intraobserver discrepancies of 10% to 20% for biopsy samples [2, 3]. Therefore, many noninvasive markers of fibrosis available via laboratory tests have been reported, and hepatitis C virus- (HCV-) positive patients have provided a good research base in this context.

It is known that significant differences are observed between HCV-positive patients and hepatitis B virus- (HBV-) positive patients, not only in the etiology, but also in terms of many other clinical parameters, including the natural history of the disease, the laboratory parameters, and the liver histology [4, 5]. However, the number of reports regarding fibrosis markers for HBV-positive patients is much lower than that for HCV-positive patients. In particular, there have been few reports about the relationship between the metabolic parameters and histological degree of liver fibrosis in HBV-positive patients, despite the fact that the liver functions as an important metabolic organ.

The values of glycated proteins reflect the plasma glucose level, and glycated hemoglobin (HbA1c) is commonly used as a reliable index of glycemic control in diabetic patients [6, 7]. The turnover period of hemoglobin in erythrocytes is about four months, and the HbA1c level therefore reflects the plasma glucose levels for the past few months [8]. Glycated albumin (GA) is another marker of the glycemic control during the past few weeks, because the turnover of albumin is about three weeks [9, 10]. In patients with CLD, hypersplenism abbreviates the lifespan of erythrocytes, leading to lower HbA1c values relative to the degree of glycemia. In contrast, the GA levels in CLD patients are higher than those estimated based on the levels of glycemia, because the turnover of serum albumin in CLD patients is increased as a result of the compensation for the decreased albumin production in the liver [11]. Since the HbA1c shows lower values and the GA shows higher values in CLD patients, the GA/HbA1c ratio is predicted to be high in patients with CLD. Indeed, the GA/HbA1c ratio has been reported to be associated with the histological stage of liver fibrosis and portal hypertension in HCV-positive CLD and nonalcoholic steatohepatitis [12–15]. In the present study, we investigated the GA/HbA1c ratio in HBV-positive patients and its correlation with liver fibrosis.

## 2. Materials and Methods

### 2.1. Patients

We studied a total of 173 HBV-positive patients who had undergone percutaneous liver biopsies between January 2008 and March 2010 at our institution. This study was retrospective and consecutively included all patients who fulfilled the following conditions: (1) HBV infection diagnosed by positive HBsAg status for at least six months. (2) Blood samples, including samples for an analysis of the GA and HbA1c levels, were obtained on the same day as the liver biopsies. Patients with the following conditions were excluded from the study: the presence of other liver diseases, hepatocellular carcinoma, immunosuppressive therapy, HCV coinfection, and insufficient liver tissue for the staging of fibrosis (a minimum of 15 mm of liver tissue with five or more portal tracts was required for diagnosis). The present study did not include patients whose GA/HbA1c ratios could have been influenced by poorly controlled diabetes.

The characteristics of the study population are summarized in Table 1. The study conformed to the ethical guidelines of the Declaration of Helsinki, and written informed consent regarding the liver biopsy and use of clinical data was obtained from all patients on admission. This study was approved by the ethics committee of the institutional review board.

### 2.2. Laboratory Data and Liver Biopsy

The HbA1c was measured by high-performance liquid chromatography, with calibration using Japan Diabetes Society (JDS) Lot 2 [15, 16]. The value for HbA1c (%) was estimated as a NGSP equivalent value (%) calculated using the following formula: in the range of JDS values ≤4.9%: NGSP (%) = JDS (%) + 0.3% and in the range of JDS 5.0–9.9%: NGSP (%) = JDS (%) + 0.4% [17]. Routine laboratory studies, including platelet counts, the prothrombin time (PT) percentage, and liver function tests (ALT, AST, alkaline phosphatase, albumin, and cholinesterase), were also performed.

In the present study, the values of two biomarkers associated with the progression of liver fibrosis (FIB-4 and the APRI, the AST-to-platelet count ratio index) were calculated, because these markers were previously shown to be associated with the progression of liver fibrosis [18–20]. The FIB-4 and APRI values were calculated based on formulas developed by Vallet-Pichard et al. [21] and Wai et al. [22], respectively: FIB-4 = Age [years] × AST [U/L]/(platelets [10^9^/L] × (ALT [U/L])^1/2^), in which the age of the patient is the age at the time of liver biopsy and APRI = 100 × (AST level/upper limit of normal)/platelets [10^9^/L].

Liver biopsy examinations were carried out according to the standard techniques. All liver samples were evaluated by well-trained pathologists at our institute, with an evaluation of the fibrosis stage and activity grade. Fibrosis was staged on a scale of F0–F4 (F0, no fibrosis; F1, portal fibrosis without septa; F2, portal fibrosis with rare septa; F3, numerous septa without cirrhosis; F4, liver cirrhosis) according to the METAVIR scoring system [23]. The histological findings of the biopsy tissues were also routinely evaluated in our department. All authors participated in the conferences about the histological findings, and the final results were confirmed by two authors (H. Enomoto and H. Imanishi) who received training for histological studies.

### 2.3. Statistical Analysis

In the present study, we investigated whether the GA/HbA1c ratio is associated with the degree of liver fibrosis in HBV-positive patients. The data for the comparisons among the groups “F0-1 versus F2 versus F3 versus F4” was analyzed by a nonrepeated measurements ANOVA, and statistical significance was consequently evaluated with the Bonferroni correction. The relationships between the GA/HbA1c ratio and other variables, including the FIB-4 and APRI, were evaluated with Spearman's correlation coefficient. A value of *P* < 0.05 was considered to be significant.

## 3. Results

### 3.1. The GA/HbA1c Ratio Increases with the Histological Stage of HBV-Positive Patients

**Figure 1:**
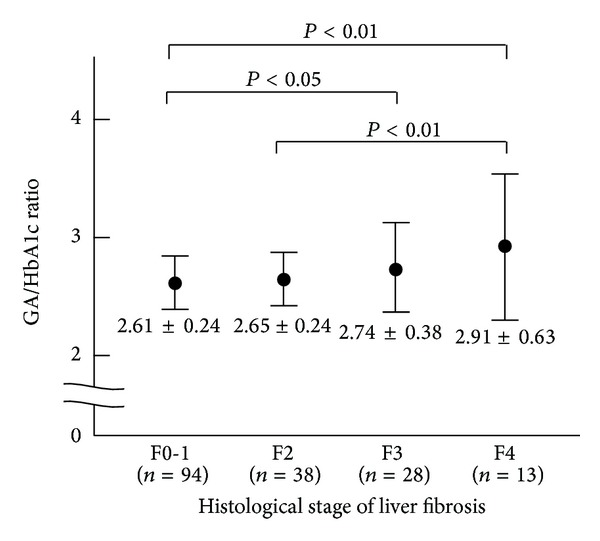
GA/HbA1c ratios in relation to the METAVIR fibrosis scores among the HBV-positive patients. The GA/HbA1c ratio increased in association with the stage of liver fibrosis. There were significant differences between the F0-F1 versus F3, F0-F1 versus F4, and F2 versus F4 groups.

A total of 173 HBV-positive patients were included in this study. The characteristics of the study population are summarized in Table 1. The population consisted of 96 (55.5%) male patients and 77 (44.5%) female patients, and the age of patients ranged from 25 to 79 years old (median 46 years old). As shown in Figure 1, the mean value of the GA/HbA1c ratio increased in association with the histological stage of liver fibrosis in the HBV-positive patients.

### 3.2. The GA/HbA1c Ratio Is Associated with the Laboratory Parameters in HBV-Positive Patients

We next investigated whether the GA/HbA1c ratio was related to the laboratory parameters of the liver function, including the PT (%), platelet count, albumin level, and cholinesterase level.  Table 2 shows that there is a significant reciprocal correlation of the GA/HbA1c ratio with the PT (%) (*R* = −0.396, *P* < 0.0001) and platelet count (*R* = −0.421, *P* < 0.0001) in HBV-positive patients. The GA/HbA1c ratio was also inversely correlated with the serum albumin level (*R* = −0.332, *P* < 0.0001) and the cholinesterase level (*R* = −0.411, *P* < 0.0001). These findings showed that the GA/HbA1c ratio increased in association with changes in the levels of markers related to liver fibrosis.

### 3.3. The GA/HbA1c Ratio and Fibrosis-Related Markers in HBV-Positive Patients

**Figure 2:**
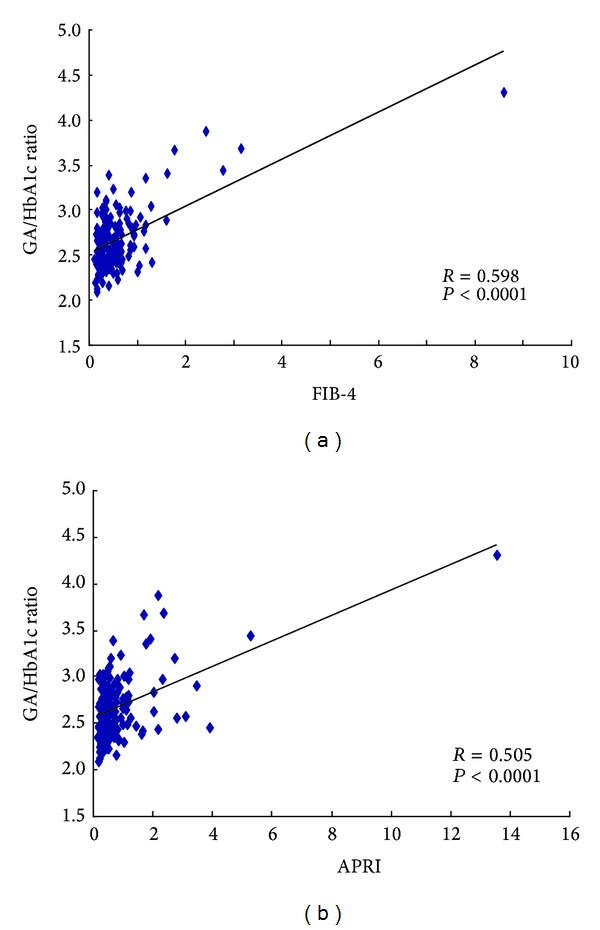
The correlation of the GA/HbA1c ratio with other fibrosis-related biomarkers. The GA/HbA1c ratio was correlated with the FIB-4 and APRI. APRI: AST-to-platelet ratio index.

Since we found that the GA/HbA1c ratio was associated with the stage of liver fibrosis in the HBV-positive patients, we therefore investigated the relationships of the GA/HbA1c ratio with two previously established fibrosis-related markers, FIB-4 and APRI. As shown in Figure 2, the GA/HbA1c ratio was significantly correlated with the FIB-4 (*R* = 0.598, *P* < 0.0001) and APRI (*R* = 0.505, *P* < 0.0001). We examined the correlations of these biomarkers with four parameters of liver function (PT percentage, albumin value, platelet count, and cholinesterase value) and found that the GA/HbA1c ratio showed better correlations than the FIB-4 value for two parameters (PT percentage and cholinesterase value). In addition, the correlations of the GA/HbA1c ratio with the findings of liver function tests were higher than those of the APRI for all four parameters (Table 2).

## 4. Discussion

Liver biopsy is the gold standard method for histologically assessing liver fibrosis. However, a liver biopsy is an invasive procedure carrying a small risk of severe complications. In addition to the FIB-4 and APRI, noninvasive biomarkers, such as the FibroTest score [24], Forns score [25], Hepascore [26], FibroMeter [27], FibroIndex [28], and Lok index [29], were previously reported to be associated with the liver fibrosis. In the present study, we showed that the GA/HbA1c ratio is associated with the histological stage of liver fibrosis in HBV-positive patients (Figure 1). We also showed that the GA/HbA1c ratio was significantly related to the laboratory variables of liver function (Table 2). Among the previously reported biomarkers for liver fibrosis, the FIB-4 and APRI are simple and useful markers that can be measured using routinely available clinical parameters without any specialized equipment. We found that the GA/HbA1c ratio significantly correlated with these well-established markers in HBV-positive patients (Figure 2). These findings suggest that there is a strong relationship between the GA/HbA1c ratio and the levels of fibrosis-related markers in HBV-positive patients.

When we examined the four variables of liver function, the correlation coefficients of the GA/HbA1c ratio were higher than those of the FIB-4 for two of the variables. In addition, the GA/HbA1c ratio was better correlated with all four variables examined than was the APRI (Table 2). It has been reported that the etiology of CLD influences the performance of liver fibrosis biomarkers. Unlike that observed in HCV-positive patients, noninvasive biomarkers are sometimes reported to not provide a correct evaluation of the degree of liver fibrosis in HBV-positive patients [30–32]. One major reason could be that the previously established biomarkers are obtained using calculations which include the AST and/or ALT. HBV infections sometimes show an acute liver inflammatory phase, and the AST and ALT values can therefore change from a mildly elevated level to an extremely high level in the same patient depending on the time when the patient is evaluated. In the present study, we included all HBV-positive patients without setting an upper limit for the AST and ALT levels, and some patients with remarkably elevated AST and ALT levels showed very high FIB-4 and APRI indices. The APRI is calculated using only the AST value and platelet count, while the FIB-4 calculation includes both AST and ALT values. Therefore, the acute elevation of AST and ALT in HBV-positive patients should more severely affect the value of the APRI than the FIB-4, although the ALT value was used as the (ALT)^1/2^ for the FIB-4 calculation. The advantage of using the GA/HbA1c ration may therefore depend on the instability of AST and ALT values in HBV-positive patients, because the GA/HbA1c ration is calculated using only the values of two glycated proteins and is independent of the AST and ALT values.

Since the liver plays a central role in metabolism, the progression of liver disease should lead to changes in metabolic parameters. However, most of the established biomarkers for liver fibrosis depend on only nonmetabolic parameters, such as the values of AST, ALT, and the platelet count. Recently, some groups, including our group, reported that the GA/HbA1c ratio was associated with the degree of liver fibrosis in various types of CLD, such as HCV-related CLD and nonalcoholic steatohepatitis [12–15]. Furthermore, we have reported that the amino acid imbalance was associated with the degree of liver fibrosis and the severity of esophageal varices in HCV-positive patients, thus suggesting that metabolism-related parameters could be potential biomarkers for the severity of CLD [33].

We herein demonstrated that the GA/HbA1c ratio increased in association with the stage of liver fibrosis in HBV-positive patients; however, the differences among the fibrosis stages were relatively small (Figure 1). Therefore, the GA/HbA1c ratio alone is not an ideal biomarker to evaluate liver fibrosis, although its correlations with the liver functional tests were as good as the previously reported well-established markers, the FIB-4 and APRI (Table 2). In addition, the present study was a simple descriptive study and did not have a prospective or longitudinal design. Therefore, we cannot draw any conclusions regarding the relationships with the progression of liver fibrosis or clinical outcomes. Recently, there was a report that it was possible to predict portal hypertension using three metabolic parameters [34]. A new biomarker based on a combination of metabolic parameters that includes the GA/HbA1c ratio would be useful for evaluating liver fibrosis in HBV-positive patients.

## 5. Conclusion

In conclusion, we herein demonstrated that the GA/HbA1c ratio increases in association with the stage of liver fibrosis and is correlated with the levels of markers related to liver fibrosis in HBV-positive patients.

## Figures and Tables

**Table 1:** The characteristics of the 173 hepatitis B virus- (HBV-) positive patients.

Age (years)	46 (25–79)
Gender (male/female)	96/77
AST (IU/L)	27 (11–269)
ALT (IU/L)	28 (7–680)
*γ*-GTP (IU/L)	25 (7–349)
ALP (IU/L)	203 (71–835)
Total bilirubin (mg/dL)	0.8 (0.1–2.3)
Albumin (g/dL)	3.90 ± 0.40
Hemoglobin (g/dL)	13.5 ± 3.8
Platelets (×10^3^/*μ*L)	178 ± 72
PT (%)	89.8 ± 12.3
Diabetes mellitus (present/absent)	6/167
Glucose (mg/dL)	91.3 ± 13.9
Triglyceride (mg/dL)	99.0 ± 45.5
Total cholesterol (mg/dL)	177 ± 32
Body mass index	22.9 ± 4.1
HBV-DNA (log⁡copies/mL)	3.7 (n.d–over 9.1)*
HBe antigen (positive/negative)	59/114
Treatment with NAs (present/absent)	67/106
Histological stage of liver fibrosis (F0-1/F2/F3/F4)	94/38/28/13

n.d: not detectable; NAs: nucleoside/nucleotide analogues.

*HBV-DNA ranged from undetectable level in patients under treatment of NAs to over measurable level (9.1log⁡copies/mL) in patients without treatment.

**Table 2:** The correlations of the three biomarkers with liver function parameters.

	Correlation coefficient		
	FIB-4	APRI	GA/HbA1c
Prothrombin time (%)	−0.362	−0.284	−0.396
Platelet count	−0.532	−0.372	−0.421
Albumin value	−0.372	−0.301	−0.332
Cholinesterase value	−0.344	−0.315	−0.411

GA/HbA1c: glycated albumin- (GA-) to-glycated hemoglobin (HbA1c) ratio.

APRI: AST-to-platelet ratio index.

## Abbreviations

GA: Glycated albumin
CLD: Chronic liver disease
HCV: Hepatitis C virus
HBV: Hepatitis B virus
PT: Prothrombin time.
